# Application of cold atmospheric plasma for decontamination of toxigenic fungi and mycotoxins: a systematic review

**DOI:** 10.3389/fmicb.2024.1502915

**Published:** 2025-01-03

**Authors:** Amanda Cristina Dias de Oliveira, Sher Ali, Carlos Humberto Corassin, Sana Ullah, Karina Nascimento Pereira, James Leon Walsh, Nataša Hojnik, Carlos Augusto Fernandes de Oliveira

**Affiliations:** ^1^Laboratory of Food Microbiology and Mycotoxicology, Department of Food Engineering, School of Animal Science and Food Engineering, University of São Paulo, Pirassununga, Brazil; ^2^School of Physics, Engineering and Technology, University of York, York, United Kingdom; ^3^Department for Gaseous Electronics, Jožef Stefan Institute, Ljubljana, Slovenia

**Keywords:** CAP technology, mycotoxins, contamination, foodstuffs, detoxificationprotect

## Abstract

**Introduction:**

Microbial contamination remains a vital challenge across the food production chain, particularly due to mycotoxins—secondary metabolites produced by several genera of fungi such as *Aspergillus, Fusarium, Alternaria*, and *Penicillium*. These toxins, including aflatoxins, fumonisins, ochratoxins, and trichothecenes (nivalenol, deoxynivalenol, T2, HT-2). These contaminants pose severe risks to human and animal health, with their potential to produce a variety of different toxic effects. Notably, up to 50% of global cereal production is affected by mycotoxin contamination, leading to significant economic losses. Current research focuses on innovative technologies to mitigate mycotoxins, with cold atmospheric pressure plasma emerging as a promising decontamination method.

**Method:**

This systematic review aimed at describing recent advances in the application of cold atmospheric plasma for the decontamination of toxigenic fungi and mycotoxins.

**Results and discussion:**

Cold atmospheric plasma offers a sustainable and cost effective solution to preserve food quality while inactivating toxigenic fungi and degrading mycotoxins. Through the generation of reactive oxygen and nitrogen species, cold plasma disrupts fungal cell integrity, hinders spore germination, and inhibits toxin biosynthesis. Additionally, cold atmospheric plasma-driven degradation of mycotoxins involves structural modifications, breaking key molecular bonds that reduce toxicity. The effectiveness of cold plasma depends on operational parameters and the specific characteristics of the treated food, with notable efficacy in degrading aflatoxin B_1_ and deoxynivalenol by converting them into less toxic substances and inhibiting their spores and DNA responsible for their biosynthesis. While the data demonstrates that cold atmospheric plasma has minimal impact on food composition, further research is needed to fully assess the nature of the degradation products of mycotoxins, its influence on food quality attributes and to optimize application strategies for different products.

## 1 Introduction

Microbial contamination is a major concern throughout the food supply chain, posing significant challenges to food production (Khaneghah et al., 2019). Among microbial contaminants, filamentous fungi are found on various food and feed products and are capable of producing toxic secondary metabolites known as mycotoxins, which present a significant threat to human health. The most common mycotoxigenic fungi include genera such as *Aspergillus*, *Fusarium*, *Alternaria*, and *Penicillium* (Khaneghah et al., 2019; Nesic et al., 2021).

Mycotoxin contamination remains a persistent challenge in agriculture, especially in cereal grains, and can occur during pre- and post-harvest stages, as well as during processing, packaging, and storage of products (Ali et al., 2023a). The most common mycotoxins found in food and feed products include aflatoxins (AFs), fumonisins (FBs), ochratoxins (OTs), zearalenone (ZEN), patulin (PAT), and trichothecenes such as nivalenol, deoxynivalenol (DON), HT-2 and T-2 toxins (Nesic et al., 2021). *Fusarium* mycotoxins, such as DON, FBs, and ZEN, are known to contaminate grains in the field during the pre-harvest stage of crop cultivation. In contrast, AFs produced by *Aspergillus* and OTs produced by *Penicillium* may appear later due to improper post-harvest practices, including inadequate storage and transport (Neme and Mohammed, 2017). The growth of mycotoxigenic fungi and the production of mycotoxins are influenced by increased temperature and moisture. As a result, higher levels of mycotoxin contamination are strongly correlated with global climate change, which also impacts the global economy. Food transported over long distances is exposed to varying local climates, extended transport, and prolonged storage times, all of which contribute to increased contamination risks (Moretti et al., 2019). It is estimated that 25–50% of global cereal production is affected by significant mycotoxin levels, with 5–10% reaching concentrations considered irremediable (Haque et al., 2020; Abrunhosa et al., 2016). Such contamination not only compromises product quality but also poses serious safety risks and is therefore subject to strict regulatory standards (Khaneghah et al., 2019). Recently, there has been increased focus on so-called emerging mycotoxins, which are either masked or modified forms of already identified types or have been newly discovered. These toxic compounds are not yet regulated but, like other mycotoxins, can occur frequently and at high concentrations in food and feed. Emerging mycotoxins include *Fusarium* metabolites such as enniatins (ENNs), fusaproliferin (FP), and beauvericin (BEA), as well as *Alternaria* spp. mycotoxins and ergot alkaloids produced by *Claviceps* spp (Gruber-Dorninger et al., 2017).

When mycotoxin levels exceed the recommended limits (EFSA, 2004; EU, 2023), they can cause acute or chronic toxic effects, with carcinogenicity being one of the most concerning outcomes (Richard, 2007). From a risk perspective, several mycotoxins have been classified based on their carcinogenic potential by the International Agency for Research on Cancer (IARC, 2023). Aflatoxin B_1_ (AFB_1_) is categorized as Group 1 (carcinogenic to humans), fumonisin B_1_ (FB_1_) and ochratoxin A (OTA) as Group 2B (possibly carcinogenic to humans), while ZEN and DON are placed in Group 3 (not classifiable as to their carcinogenicity to humans) (IARC, 2023; Schrenk et al., 2020; Ostry et al., 2017; EFSA, 2004). In addition to genotoxicity and carcinogenicity, mycotoxins are known to cause a range of other toxic effects. Human exposure, particularly in young children, is especially concerning due to their immature detoxification systems and increased vulnerability to these toxins (Knutsen et al., 2017; Fakhri et al., 2019). Exposure to FB_1_ has been linked to neural tube defects and cancer (Alvito and Pereira-da-Silva, 2022; Schrenk et al., 2022), while ZEN is known to cause estrogenic disturbances and degenerative syndromes, affecting both growth and reproductive health in humans and animals (Borutova et al., 2012; Schoevers et al., 2012). The T-2 toxin can inhibit protein, RNA, and DNA synthesis, leading to apoptosis, necrosis, lipid peroxidation, and adverse hematopoietic effects (Arcella et al., 2017). DON disrupts eukaryotic cells and mitochondria, with chronic exposure linked to intestinal microbiota imbalance and health complications (Knutsen et al., 2017; Ali et al., 2024; Arce-López et al., 2021; Akbari et al., 2017). OTA is recognized for its nephrotoxic, neurotoxic, teratogenic, and carcinogenic properties (Clark and Snedeker, 2006; Majeed et al., 2017).

Preventive measures including good agriculture practices, appropriate grain handling after harvest and proper storage are key factors to avoid fungal development and mycotoxin production (Haque et al., 2020). However, these practices may not be effective once mycotoxins have formed. Therefore, efforts have been directed toward developing effective strategies to alleviate or eliminate toxins and their metabolites from food materials (Neuenfeldt et al., 2023). Effective decontamination approaches must minimize both toxigenic fungi and mycotoxins levels, without compromising food quality and safety. So far, various methods—including chemical, biological, physical—have been explored in the food industry for mycotoxin detoxification (Marshall et al., 2020; Basso et al., 2023; Ali et al., 2023b; Naeem et al., 2024). Chemical treatments, such as ammonization, ozone, hydrogen peroxide, and sodium bisulfite among others, have been widely applied to detoxify mycotoxins in various food matrices. However, these methods may reduce nutritional quality, leave harmful residues, or only partially remove toxins, raising concerns over safety and consumer acceptance (Nahle et al., 2022; Mir et al., 2021; Khan, 2024). Biological methods involving microorganisms or enzymes to adsorb or degrade toxins offer safer alternatives but can face challenges with production consistency (Loi et al., 2023; Mir et al., 2021). Physical methods, such as thermal treatments, can effectively reduce microbial loads and toxins but may alter sensory and nutritional properties, or achieve uneven heating in grains (Naeem et al., 2024; Anderson, 2019). In contrast, innovative approaches to mycotoxin detoxification such as non-thermal technologies are anticipated to have a pivotal role in the food supply chain, ensuring the preservation of food quality while minimizing environmental impacts and maintaining economic viability (Hojnik et al., 2020). Recently, research has increasingly focused on cold atmospheric pressure plasma (CAP) as a promising approach for mycotoxin decontamination of food, aligning with these critical requirements (Patriarca and Pinto, 2017; Gavahian et al., 2021). CAP has demonstrated effectiveness in reducing microbial loads and detoxifying mycotoxins across diverse food types, especially in low-moisture products like cereal grains (Sun et al., 2014; Mir et al., 2021; Naeem et al., 2024). Although some studies have outlined CAP applications for fungi and mycotoxin decontamination (Gavahian and Cullen, 2019; Neuenfeldt et al., 2023; Misra et al., 2018; Hojnik et al., 2017), there is no available systematic reviews on this subject. Therefore, the objective of the present study was to systematically review recent literature on the application of CAP for the decontamination of commonly occurring mycotoxins in foodstuffs, discussing its potential and challenges for broader adoption in the food industry.

## 2 Search strategy

A literature search was conducted according to Cochrane protocols and the preferred reporting items for systematic review and meta-analysis (PRISMA) guidelines (Shamseer et al., 2015) across PubMed, ScienceDirect, Web of Science, Scopus and Google Scholar to identify relevant studies on the use of CAP for decontamination of main mycotoxins found in food products published in the past 10 years. The following key terms were employed: “Mycotoxins” OR “Aflatoxins” OR “Fumonisins” OR “Ochratoxin A” OR “Zearalenone” OR “Patulin” OR “Trichothecenes” OR “Deoxynivalenol” OR “T-2 Toxin” OR “HT-2 Toxin” OR “Atmospheric pressure plasma technology” OR “Cold atmospheric plasma” OR “Decontamination” OR “Detoxification” OR “Degradation.”

To optimize search precision and account for database-specific limitations, the terms were grouped into five search combinations:

1. “Cold atmospheric plasma” AND “Mycotoxins” AND “Aflatoxins” OR “Deoxynivalenol” AND “Degradation.”
2. “Atmospheric pressure plasma technology” AND “Mycotoxins” OR “Fumonisins” OR “Patulin” AND “Decontamination.”
3. “Cold atmospheric plasma” AND “Ochratoxin A” OR “Zearalenone” AND “Degradation” OR “Detoxification.”
4. “Atmospheric pressure plasma technology” AND “Mycotoxins” OR “Trichothecenes” OR “Toxin HT-2” AND “Detoxification.”
5. “Cold atmospheric plasma” AND “Mycotoxins” OR “Toxin T-2” OR “Trichothecenes” AND “Decontamination.”

**FIGURE 1:**
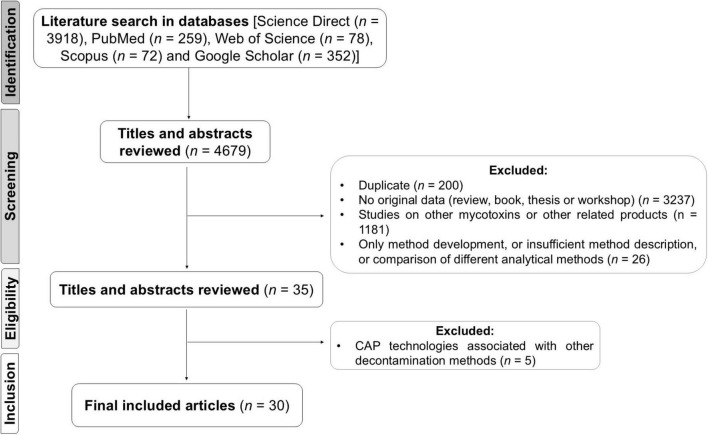
Flow diagram of literature search, inclusion and exclusion criteria, and data collection.

After screening titles and abstracts for relevance, full-text articles were assessed for eligibility based on the following criteria: (1) availability of full text, (2) original research studies (excluding reviews), (3) detailed experimental procedures, (4) precise analytical methods and (5) publication in English. The initial search yielded 4,679 articles, from which 3,237 articles were excluded immediately for not meeting criterion 2, as they were not original studies. Additionally, 200 articles were removed as duplicates, resulting in a total of 1,242 unique publications on the application of CAP. Following another screening, 1,181 articles were excluded for describing CAP applications for secondary fungal metabolites other than main mycotoxins that commonly occur in food products or involving products unrelated to the food sector. Further 26 articles were excluded due to incomplete descriptions of the methods used or because they focused on comparisons between different analytical methods. Finally, 5 articles that used CAP in combination with other decontamination methods were also excluded. At the end of the screening process, a total of 4,650 articles were excluded, as outlined in Figure 1. Ultimately, 30 studies met the inclusion criteria and were included in this review.

## 3 Principles of cold atmospheric plasma technologies

Plasma, known as the 4th state of matter, is created when sufficient energy, in the form of heat or an electric field, is applied to a gas, causing free electrons to be accelerated to high energies. Collisions between electrons and neutral gas atoms or molecules can result in ionization, if a sufficient number of ionization events occur the process becomes self-sustaining and a plasma is formed (Neuenfeldt et al., 2023). Atmospheric pressure plasmas can be categorized as either “thermal,” where a local thermodynamic equilibrium is maintained among the electrons and heavy particles (e.g., ions and neutrals), or “non-thermal,” characterized by thermodynamic imbalance, whereby electrons are at a significantly higher temperature. CAP falls within the non-thermal category (Pankaj and Keener, 2017).

Two essential components are required for plasma generation: a high-voltage source and a reactor. The high-voltage source is used to establish a high electric field, which accelerates electrons to the high energies needed to cause ionization, this is typically done in a reactor employing two electrodes, a powered electrode and ground (Niemira, 2012; Ojha et al., 2021). Key parameters affecting the efficacy of the system relate to the form of the applied voltage (e.g., direct current, radio-frequency, pulsed, microwave etc.), and the particular configuration of the reactor (e.g., plasma jet, parallel plate etc.). Once plasma forms in the reactor, the energetic electrons take part in a plethora of reactions causing dissociation and excitation of the background gas creating a complex mixture including ions, free radicals, electrons, and UV photons. For example, in humid air plasma it is known that over 50 unique chemical species are created, which take part in over 600 reactions (Sakiyama et al., 2012). Of particular importance are the reactive neutral species, commonly termed reactive oxygen species (ROS) and reactive nitrogen species (RNS) (Hertwig et al., 2018; Misra and Jo, 2017). Beyond the key physical parameters of the plasma system, a large number of factors affect ROS and RNS generation and therefore the efficacy of the plasma in degrading mycotoxin contamination. For example, plasma generation parameters, such as energy input, play a key role, as the electrical power used for plasma generation directly affects the energy available for the chemical ionization, dissociation, and excitation processes. These energy-dependent processes determine the types and densities of reactive species formed (Shimizu et al., 2012). The energy distribution of electrons is another vital factor, as it governs the nature of reactions within the plasma. Specifically, the electron energy distribution function (EEDF) influences whether molecular dissociation or ionization occurs (Abdel-Fattah, 2013). The frequency of the applied voltage, whether direct current (DC), radio frequency (RF), or microwave, also impacts the energy distribution of electrons and ions. Abdel-Fattah (2013) demonstrated that higher frequencies led to increased peak electron densities, a finding supported by Naidis (2014), who observed elevated densities of reactive species at higher voltage amplitudes and frequencies. In a helium-air plasma model, Naidis (2014) noted that higher voltage amplitudes and frequencies increased NO and O3 densities while reducing O atom concentrations, whereas the hydroxy radical (OH) density remained relatively stable.

The feed gas composition is another critical determinant of plasma chemistry, as it defines the atoms and molecules participating in plasma formation. Variations in feed gases, such as helium (He), argon (Ar), nitrogen (N_2_), air, or oxygen (O_2_), result in distinct reactive species profiles (Naidis, 2014; Han et al., 2016; Dünnbier et al., 2013). The introduction of specific gases can significantly influence reactive species generation. For example, Han et al. (2016) observed a gradual increase in hydrogen peroxide (H_2_O_2_) production with the addition of oxygen, a process linked to enhanced generation of ROS, including OH radicals. Gas flow rate also affects the formation and distribution of reactive species such as NO and HO_2_. Increased flow rates promote the spread of OH radicals, affecting their interaction sites (Hasan and Walsh, 2017). Interaction with ambient air further modulates plasma chemistry, introducing nitrogen, oxygen, and water vapor into the system, which contributes to RNS and ROS formation (Naidis, 2014; Morabit et al., 2021).

Electrode geometry and material significantly impact the electric field distribution, which influence plasma characteristics. Changes in electrode geometry, for instance diameter, alter the breakdown voltage and power deposition within the plasma discharge (Hasan and Walsh, 2017). The chemical composition of plasma also depends on the distance between electrodes and the target. Variations in these distances modify the ionization region, affecting the generation and transport of ROS and RNS (Morabit et al., 2021). Moreover, the density of reactive species decreases as the distance between the plasma jet and the target sample increases (Zhang et al., 2019). Plasma interactions with either a biological, liquid, or polymeric sample, can catalyze or inhibit specific reactions, altering the chemical composition near the sample surface (Morabit et al., 2021; Kovaèeviæ et al., 2018). In some types likely liquid samples, these effects are particularly pronounced, as the presence of the liquid alters reactive species composition and modifies the potential gap near the target surface (Kovaèeviæ et al., 2018).

Historically, CAP generation was constrained to low-pressure conditions, requiring the use of a vacuum chamber and thus limiting its applications for the treatment of food products. However, advancements in technology now allow for the stable and non-thermal generation of plasma at atmospheric pressure, leading to extensive research across various scientific fields (Pankaj and Keener, 2017). In specific reactor configurations, such as plasma jets, the mass transport of reactive species is facilitated by gas flow, enabling the effects of plasma to extend to regions well beyond the electrodes. Due to the limited area covered by a single plasma jet, multiple jets or motion systems are often used to treat larger areas or volumes (Feizollahi et al., 2020).

The effectiveness of CAP in fungal inactivation depends on several factors, including the type of plasma generator, the gas utilized, the fungal strains involved, and the test matrix (Mošovská et al., 2019; Wu et al., 2021). Additionally, specific equipment parameters, such as frequency (ranging from 0 Hz to several GHz), discharge voltage (1 kV to over 100 kV), and power (ranging from W to kW), significantly influence CAP’s impact (Ojha et al., 2021). While numerous studies highlight the importance of these parameters, there remains no consensus among recent research regarding the optimal ranges for CAP treatment. The variability in these parameters, as applied to mycotoxin degradation in food, is further discussed according to the specific CAP types examined in the studies.

### 3.1 Indirect plasma treatment

As previously described, a wide range of different plasma reactors have been used to examine the impact of plasma exposure on mycotoxin contamination. Typically, these can be categorized as either direct—meaning the plasma directly interacts with the target, or indirect—meaning the plasma is generated remotely from the target and only the neutral species interact with the target. This is a fundamental difference, as the spatial separation between plasma and target acts as a spatial filter for reactive species, meaning only the less reactive agents, such as ozone, play a role in the degradation process.

**FIGURE 2:**
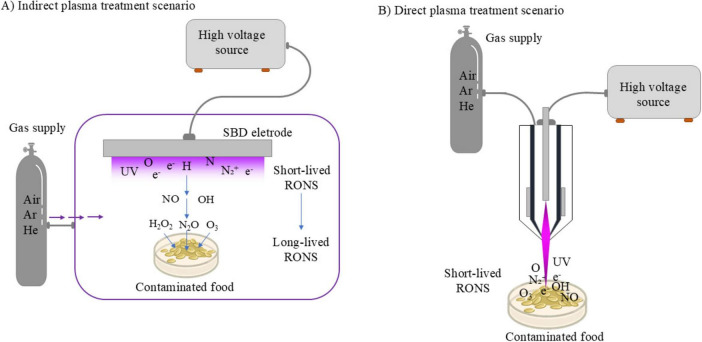
Schematic representation of indirect **(A)** and direct **(B)** plasma treatments illustrating mechanisms for decontaminating food matrices.

Of the many indirect plasma treatment systems reported in the literature, the surface barrier discharge (SBD) has been widely adopted. This particular configuration belongs to the dielectric barrier discharge (DBD) family of devices, and typically employs a dielectric material sandwiched between a powered and grounded electrode, illustrated in Figure 2A. In this indirect scenario, the plasma region is in the form of a thin layer situated mm to cm from the target. When ambient air is used as the working gas, highly reactive species such as H, N and O form in the discharge layer and are transported toward the target via diffusion and convection (Dickenson et al., 2018; Hasan et al., 2017; Dickenson et al., 2017; Hasan and Walsh, 2017). Owing to their high chemical reactivity, they rapidly react beyond the discharge region to form more stable states such as O_3_ and N_2_O. Other indirect treatment modalities include plasma jets where the target is placed at a distance from the generated plasma plume and plasma “activated” liquids (Naidis, 2014; Morabit et al., 2021). In a plasma activated liquid, either direct or indirect plasma exposure can be used to first treat a liquid, such as water, introducing a complex chemical cocktail; with the liquid being subsequently applied to the target. Typically, this process generates an acidic solution, owing to abundant nitrogen-based species whilst containing hydrogen peroxide and others. Such solutions have been shown to possess disinfectant properties, making them effective for surface decontamination (Neuenfeldt et al., 2023; Han et al., 2023; Perinban et al., 2022).

### 3.2 Direct plasma treatment

In contrast to the in-direct treatment scenario, the direct application of CAP to a target enables the entire reactive chemistry created within the discharge to play a role in the decontamination process (Figure 2B). Plasma reactors, such as plasma jets and parallel plates, facilitate the direct exposure of a target to a plasma (Harikrishna et al., 2023). Both plasma jets and parallel plates can take the form of a DBD or employ other approaches to inhibit the glow-to-arc transition, such as radio-frequency excitation (Park et al., 2000; Polito and Kushner, 2024). Through these methods it is possible to maintain discharge stability and a non-thermal characteristic. While such systems can offer enhanced efficacy over their indirect counterparts in microbial inactivation (Wan et al., 2019), due primarily to the enhanced flux and composition of RONS arriving at the target, they are also more complex and typically more difficult to control. When a target is electrically conductive, such as most food stuffs, it is inherently part of the electrical circuit and therefore plays a role in dictating the specific composition of the plasma. Plasma jets can overcome this by physically separating the plasma generation region from the application region, but typically require significant gas flows and/or operation in noble gases such as Argon and Helium (Morabit et al., 2021).

## 4 Application of cold atmospheric plasma in inactivating toxigenic fungi and mycotoxin biosynthesis

**FIGURE 3:**
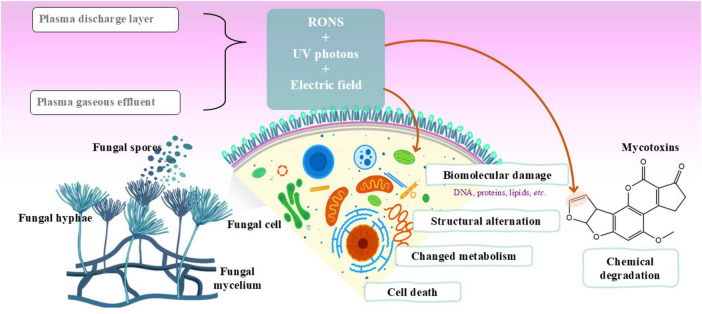
Mechanisms involved in the inactivation of toxigenic fungi and the inhibition or degradation of mycotoxins induced by cold atmospheric plasma (CAP) technology.

In foodstuffs and other substrates, fungal sporulation and germination evolve until a fully developed fungus. For this process, the fungus and its spores nurture on the target food matrix and introduce it with different chemical agents such as damaging enzymes, and self-defense metabolites including mycotoxins (Ali et al., 2023a). The fungus cellular integrity, its cellular metabolic processes and the enzymatic events are essential in establishing this cycle. However, injury to any or all the essential components, leads to an end with the fungus sporulation and germination with no more production of toxins. Inhibiting such mechanisms has been tempted with modern technologies including CAP application on certain foods. Figure 3 highlights the mechanisms underlying the inactivation of toxigenic fungi and inhibition of mycotoxin biosynthesis by CAP technologies.

The antimicrobial properties of CAP are well-established, making this technology an appealing option for controlling mycotoxigenic fungi and decontaminating the mycotoxins they produce. Table 1 summarizes the main findings from studies focused on the inactivation of toxigenic fungi and inhibition of mycotoxin biosynthesis. CAP generates different reactive species like ROS, RNS and radicals (Hertwig et al., 2018; Misra and Jo, 2017), which are capable of damaging spores and avoid their germination. To do so, the chemical species are directed to induce oxidative stress on the outer layers of the target spores. The oxidative stress further causes a disruption to the cellular metabolic events, and cellular membrane together with its essential DNA, protein, and lipid components, resulting in declined growth of fungus and a reduced production of toxins (Zhao X. et al., 2024). The exposure of *A. ochraceus* to CAP caused ruptures and desiccations in its spores, with significant decline in their viability (Hoppanová et al., 2020). Membrane rupture and desiccation allow the cellular contents to escape from the damaged cell and eventual cell death occurs. In addition, CAP can lead to poor production or lack of energy, ultimately causing spore prevention and cell death, as described for *Clostridioides difficile* (Kaur et al., 2020). CAP can also interfere in the metabolic pathways and enzyme events needed to spore development (Hojnik et al., 2017).

**TABLE 1:** Summary of main outcomes from studies using cold atmospheric pressure plasma (CAP) in decontaminating toxigenic fungi.

Fungal species	Matrix type	CAP source (process parameters)	Main outcomes	References
*Aspergillus niger*, *Rhizopus oryzae*, *Penicillium verrucosum*, and *Fusarium graminearum*	Rice grain	DBD (Distance: 20 mm; voltage: 25 kV; time: 2, 4, 6, 8 min)	↓ 65–80% spore formation, ≠ DON biosynthesis	Guo et al., 2023
*F. graminearum HX01*, *F. graminearum LY26*, *F. pseudograminearum*, and *F. moniliforme*	Spore suspensions in carboxymethylcellulose liquid medium	SMD (Frequency: 7 kHz; discharge power: 5 ± 0.15 W; distance: 3 mm; time: 0.5, 1, 1.5, 2, 2.5, 3, 3.5 min; gases: O_2_ and N_2_; indirect exposure)	(I). *F. moniliforme* ↓ at 6.0 log_10_, *F. graminearum HX01* ↓ at 5.1 log_10_, *F. graminearum LY26* ↓ at 2.5 log_10_, *F. pseudograminearum* ↓ at 2.0 log_10_ (II). *F. graminearum LY26* ↓ at 4.9 log_10_ CFU. mL^–1^, *F. pseudograminearum* ↓ at 3.9 log_10_ CFU. mL^–1^	Wang Y. et al., 2022
*A. niger* and *P. verrucosum*	Barley	DCSBD 400 (Power: 350 W; gas flow rate: 10 L/min; time: 1 or 3 min; gas: 100% CO_2_ + 80% CO_2_ + 20% O_2_)	(III). ↓ mold count of *A. niger* and *P. verrucosum* by 2.5–3 log cycles	Durek et al., 2018
*A. flavus*	Corn	DBD (Frequency: 45–250 Hz; voltage: 60–160 kV; distance: 30 mm; time: 4, 8, 12 min)	(IV). *A. flavus* spores ↓ by 0.96–3.20 log_10_ CFU/g (V). AFB_1_ produced by *A. flavus* ↓ by 96.16%	Zhao L. et al., 2024
*A. flavus*	*A. flavus* spore suspension in sterile water	DBD (Voltage: 50, 60, 70 V; distance: 6 mm; time: 0, 1, 2, 3, 4, 5, 6 min)	(VI). 88% ≠ for *A. flavus*, ↓ *A. flavus* spores by 4.47 log_10_ CFU/mL	Zhao L. et al., 2024
*A. flavus*	Fungal malt extract agar with *A. flavus* spores	HVCAP (Frequency: 50 Hz; voltage: 70, 80, 85 kV; time: 1, 2, 5, 10 min; gases: O_2_, N_2;_ direct exposure)	50% ≠ of *A. flavus* spores, ↓ > 99% of DON (in aqueous suspension), ↓ 33% of DON (in powdered form)	Ott et al., 2021
*A. flavus* and *A. parasiticus*	Wheat grains	Gliding arc cold plasma [Frequency: 20 kHz; power: 5–10 W; gases: dry air (21% O_2_, 79% N_2_); time: 2, 6, 12 min]	57% ↑ in lag time, 68% ↓ in growth rate and 78% ↓ of *A. flavus* 70% ↑ in lag time, 55% ↓ in growth rate and 68% ↓ of *A. parasiticus*	Rahnavard et al., 2024
*A. niger*	*A. niger* spore suspension from contaminated date palm fruits	Plasma jet (Voltage: 25 kV; frequency: 25 kHz; dist.: 12 mm; time: 7.5 min; gas: argon; indirect exposure)	*A. niger* spores ↓ from 1000 CFU/100 mm^2^ (control) to 20 CFU/100 mm^2^	Ouf et al., 2014
*A. niger*	Spore suspension of *A. flavus* cultured on PDA	DB (Frequency: 45–250 Hz; voltage: 60–160 Kv; distance: 14 mm; time: 2 min; gas: O_2_, N_2_; direct exposure)	(VII) ↓ 5.39 Log_10_ CFU/cm^2^, 17–66% ≠ in *aflE* and *aflM* genes	Zhao et al., 2023

CFU/g, colony-forming unit per gram; CFU/mL, colony-forming unit per milliliter; DBD, dielectric barrier discharge; DCSBD, diffuse coplanar surface barrier discharge; I: results obtained with 3 min time of exposure; II: results obtained with 3.5 min time of exposure; III: results obtained with 3 min time of exposure; IV: results obtained with 8 min time of exposure; V: results obtained with 12 min time of exposure; VI: results obtained with 6 min exposure time of plasma at 60 V; VII: results obtained with 2 min time of exposure; SDBD, surface dielectric barrier discharge; ↓ reduction; ≠ inhibition or inactivation; ↑ increase. NI, not informed.

The inhibition efficacy of CAP created with DBD was evaluated in a case of decontaminating *A. niger*, *P. verrucosum*, *F. graminearum*, and *Rhizopus oryzae* on rice grains (Guo et al., 2023). Promising results were achieved after 4 min of exposure, significantly reducing fungal mycelial growth across all tested species, with increased efficacy observed at a longer exposure time (Table 1). The study demonstrated a significant inhibitory effect on spore germination, particularly in the case of *F. graminearum* and *R. oryzae*, for which a 4-min exposure resulted in a 65–80% reduction in spore viability (Guo et al., 2023). However, the efficiency of CAP against spores varies according to the fungal genus and spore size (Wang Y. et al., 2022; Zhao X. et al., 2024). The larger spore sizes are shown to correlate well with a lower CAP inactivation efficiency (Wen et al., 2017; Wen et al., 2019; Wen et al., 2020). By studying the inactivation ability of CAP on *Fusarium* species, Wang Y. et al. (2022) found that smaller sized spores were more susceptible to CAP reactive species due to their larger specific surface area, resulting in more effective inactivation. Additionally, spore germination rates dropped below 10% following to 2 min of exposure to CAP generated with a dielectric barrier surface micro-discharge (SMD). Regarding DON levels relative to the dry weight of mycelium (μg/g), CAP treatment resulted in a 30–48% reduction (Wang Y. et al., 2022).

CAP’s action can alter spore morphology, leading to structural damage such as increased roughness surface, membrane fractures, and size reduction. The observed membrane damage, confirmed by monitoring intracellular nucleic acid and protein leakage, indicates that increasing CAP exposure beyond 2 min progressively compromises membrane integrity (Wang Y. et al., 2022). CAP also inhibits mycotoxin biosynthesis by downregulating the expression of the genes responsible for toxin production. Ouf et al. (2014) found that CAP generated with a double atmospheric pressure argon plasma jet (DAPACP) or plasma jet affected the biosynthesis of FB_2_ and OTA in treated *A. niger* spores, through disruptions in the microbial DNA. This was also investigated in a study by Zhao et al. (2023), where aflatoxin production was analyzed at the biomolecular level, focusing on the gene expressions involved in aflatoxin biosynthesis. Among the 12 genes associated with AFB_1_ production, CAP generated with the dielectric barrier (DB) specifically reduced the expression of *aflE* and *aflM*, indicating CAP’s role in inhibiting aflatoxin synthesis. These findings highlight the importance of advancing our understanding of CAP’s impact on fungal activity and mycotoxin production at the molecular level.

## 5 Mycotoxins degradation by cold atmospheric plasma

**FIGURE 4:**
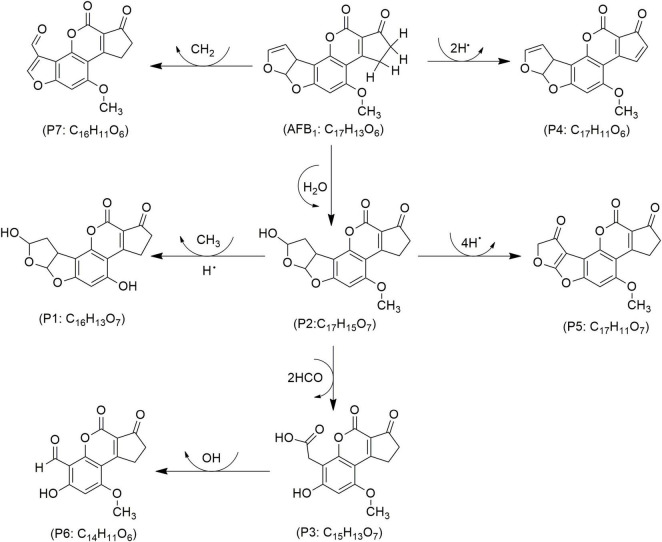
Proposed mechanisms and degradation products (P) of aflatoxin B_1_ (AFB_1_) in food under cold atmospheric plasma (CAP) treatment, adapted from Zhao et al. (2023).

**FIGURE 5:**
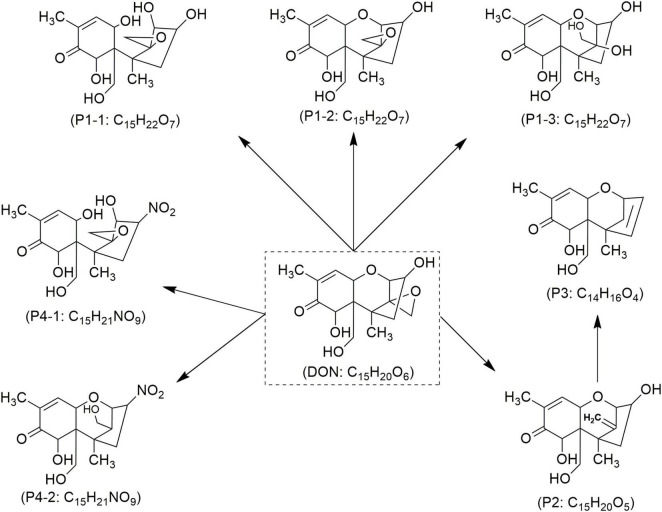
Proposed mechanisms and degradation products (P) of deoxynivalenol (DON) in food under cold atmospheric plasma (CAP) treatment, adapted from Chen et al. (2022).

**FIGURE 6:**
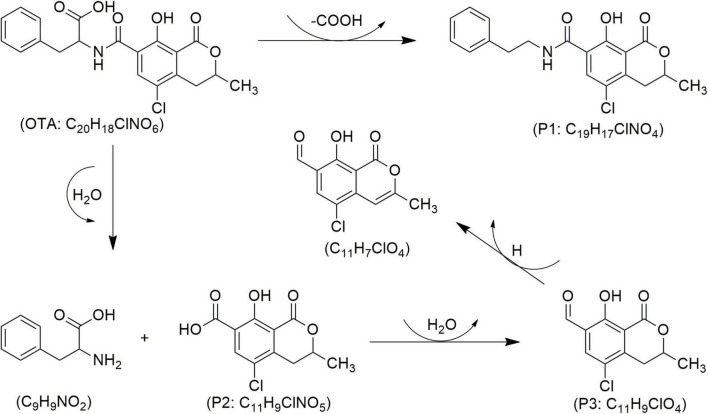
Proposed mechanisms and degradation products (P) of ochratoxin A (OTA) in food under cold atmospheric plasma (CAP) treatment, adapted from Wang et al. (2024).

**FIGURE 7:**
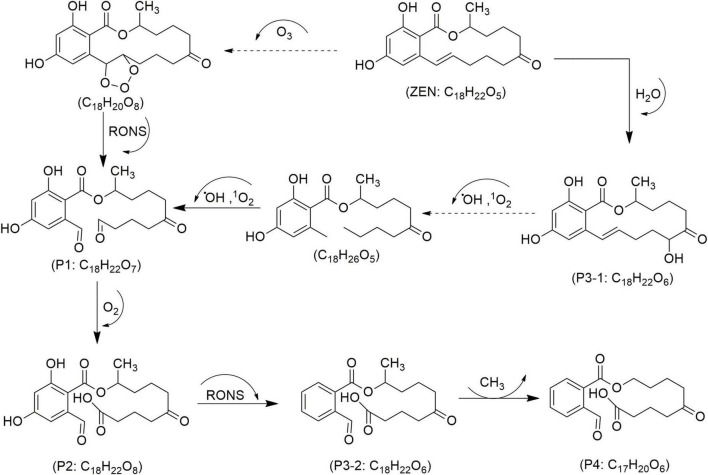
Proposed mechanisms and degradation products (P) of zearalenone (ZEN) in food under cold atmospheric plasma (CAP) treatment, adapted from Liu et al. (2024).

**FIGURE 8:**
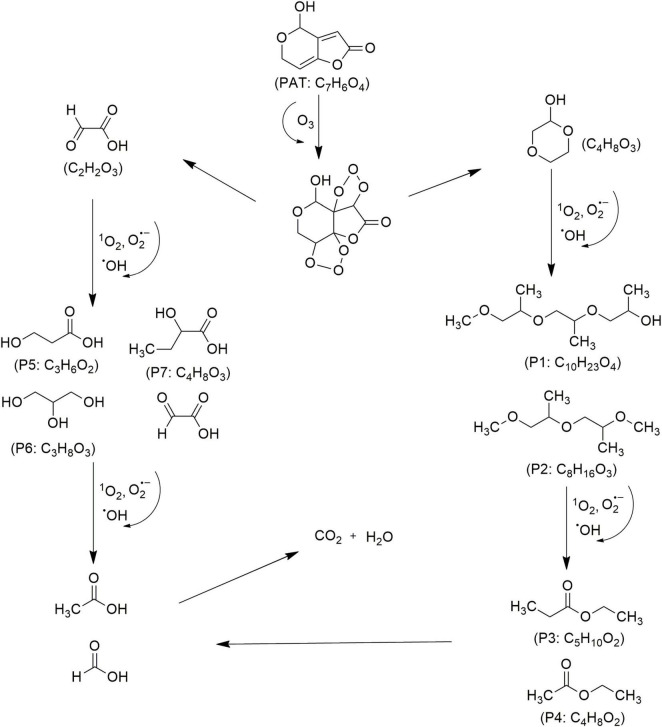
Proposed mechanisms and degradation products (P) of patulin (PAT) in food under cold atmospheric plasma (CAP) treatment, adapted from Shirazi et al. (2025) and Xue et al. (2021).

Mycotoxin reduction through the application of CAP technologies occur mainly by degradation where a target toxin is broken down into less or non-toxic chemical components (Wang et al., 2024). Mycotoxins’ degradation through CAP is primarily influenced by the type of chemical reactions, the reactive species generated by the apparatus, and UV radiation. This combination leads to cleavage of the molecular structure of toxin, resulting in the formation of transformation products (TPs) (Feizollahi et al., 2020). When CAP induces the cleavage of the specific site on the mycotoxin molecule responsible for its toxicity, it can be assumed that this process leads to the degradation of the mycotoxin and the formation of less toxic TPs. In line, reduction effects following CAP application have been demonstrated for AFs and some other mycotoxins (Figures 4–8), as shown in Tables 2, 3, respectively.

**TABLE 2:** Summary of main outcomes from studies using cold atmospheric pressure plasma (CAP) in decontaminating aflatoxins.

Aflatoxin	Initial concentration	Type of matrix	CAP source (process parameters)	Main outcomes	Degradation products	References
AFB_1_	10 μL	*In vitro* Rice and wheat (*A. flavus*)	CDPJ (Voltage: 20 kV; frequency: 58 kHz; current: 1.00, 1.25, 1.50 A; distance: 15, 25, 35 mm)	95% AFB_1_ Φ, 56% AFB_1_ ↓ (rice) 45% AFB_1_ ↓ (wheat)	NI	Puligundla et al., 2020
AFM_1_	0.1, 1, and 50 μg/L	Milk (standard solution)	HVCAP (Voltage: 80 kV; frequency: 60 Hz; power: 200 W; time: 20 min; direct and indirect exposure)	80% AFM_1_ ↓ in milk using MA 65 operating gas, 65.0% AFM_1_ ↓ in milk using air, no change in milk color	NI	Nguyen et al., 2022
AFB_1_	50 μg/mL*	Corn grain (standard solution)	SBD (Distance: 5 mm; low power: 0.18 W/cm; high power: 0.31 W/cm; time: 5, 10 min; gas: O_2_ and N_2_; indirect exposure)	100% AFB_1_ decontamination, negligible change in corn morphology	C_17_H_15_O_7_ C_16_H_11_O_6_ C_15_H_13_O_7_ C_14_H_10_O_6_	Hojnik et al., 2020
AFB_1_	0.1 μg/mL	*In vitro* Corn (standard solution)	DBD (Voltage: 6 kV; frequency: 20 kHz; distance: 12 mm; time: 10 min; gas: He, N_2_)	66% AFB_1_ ↓, No toxic by-products formation of matrix constituent	C_17_H_12_O_6_ C_17_H_14_O_8_ C_16_H_12_O_7_ C_15_H_12_O_7_ C_15_H_10_O_5_ C_15_H_10_O_7_ C_14_H_12_O_5_	Wielogorska et al., 2019
AFB_1_	0.05 μg/mL	*In vitro*	HVCAP (Voltage: 30–130 kV; frequency: 60 Hz; time: 1–5 min; gas: gas: O_2_ and N_2_; direct and indirect exposure)	77% and 93.3% ↓ in AFM_1_ (I) 56.4% and 89.6% ↓ in AFM_1_ (II)	NI	Nikmaram and Keener, 2023
AFB_1_ AFs	AFB_1_: 20 μg/kg, AFB_2_: 50 μg/kg, AGB_1_: 100 μg/kg, AGB_2_: 150 μg/kg	Rice (*A. parasiticus*)	DBD [Voltage: 28–169 kV; frequency: 50–200 Hz; distance: 35 mm; time: 5–60 min; gas: O_2_ (0–65%), N_2_ (5–70%), CO_2_ (30%); direct and indirect exposure]	55.34% ↓ in AFB_1_, 56.37% ↓ in AFs, no change in rice quality except fat oxidation, ↓ AFB_1_ (66.12–29.19 μg/kg) and ↓ AFs (96.93–208.58 μg/kg) (III)	NI	Zhi et al., 2023
AFB_1_	1 mg/L	Corn (*A. flavus*)	DBD (Frequency: 45–250 Hz; voltage: 60–160 kV; distance: 30 mm; time: 12 min)	80% Φ of AFB_1_	NI	Zhao L. et al., 2024
AFM_1_	0.05 mg/L	Milk (standard solution)	HVCAP [Frequency: 60 Hz; voltage: 30–130 kV; time: 1, 3, and 5 min; gas: O_2_ (65%), N_2_ (5%), CO_2_ (30%); direct and indirect exposure]	41.9% and 37.8% AFM_1_ ↓ in skim milk and whole milk (IV), 87% AFM_1_ ↓ with 4 h of post treatment (V)	NI	Nikmaram and Keener, 2022
AFB_1_	50 μg/mL	*In vitro*	DBD (Frequency: 10 kHz; voltage: 10 kV; distance: 30 mm; time: 1, 2, 5, 15, 30 min; indirect exposure)	75.8% ↓ after 1-min 80.9% ↓ after 2-min 81.8% ↓ after 5-min 82.3% ↓ after 15-min 82.5% ↓ after 30-min	NI	Sakudo and Yagyu, 2024
AFM_1_	10 μg/mL	*In vitro* (Petri dish contaminated with AFM1)	HVCAP [Frequency: 60 Hz; voltage: 30–130 kV; time: 1, 3, and 5 min; gas: O_2_ (65%), N_2_ (5%), CO_2_ (30%); direct and indirect exposure]	Φ of AFM_1_ (VI), ↓ in AFM_1_ bioactivity due to loss of C8–C9 double bond in furofuran ring	C_15_H_11_O_7_ C_17_H_15_O_9_ C_15_H_13_O_7_	Nikmaram et al., 2023
AFB_1_ AFB_2_ AFG_1_ AFG_2_	100 μg/kg	Pistachio kernels (standard solution)	SDBD (Frequency: 23 kHz; voltage: 6 kV; power: 42–425 W; time: 0–60 min; gas: O_3_ and NO_x_; indirect exposure)	Experiment under O_3_: ↓ in AFB_1_ (81%) and AFG_1_ (82%) on 15 min, 99% ↓ in AFB_1_ + AFG_1_ on 60 min, 60% ↓ in AFB_2_ + AFG_2_ Experiment under NO_x_: ↓ in AFB_1_ (64%), AFG_1_ (63%), AFB_2_ (17%), AFG_2_ (19%)	NI	Laika et al., 2024
AFB_1_ AFB_2_ AFG_1_ AFs	NI	Wheat grains (*A. flavus* and *A. parasiticus*)	Gliding arc cold plasma [Frequency: 20 kHz; power: 5–10 W; time: 12 min; gas: O_2_ (21%), N_2_ (79%)]	↓ in AFB_1_ (64%), AFB_2_ (41%), AFG_1_ (59%), AFG_2_ (40%), TAF (61%)	NI	Rahnavard et al., 2024
AFB_1_ AFB_2_ AFG_1_ AFG_2_	AFB_1_: 0.2 μg/mL AFB_2_: 0.2 μg/mL AFG_1_: 0.05 μg/mL AFG_2_: 0.05 μg/mL	*In vitro* (Stock solutions of mycotoxins)	SDBD (Frequency: 40 kHz; voltage: 7–10 kV; distance: 5 mm; time: 1, 2, 4, 8 min; gas: O_2_, N_2_; indirect exposure)	99% ↓ in AFB_1_, at 30 s AFB_1_ values ↓ from 18 μg/kg to < 0.006 μg/kg, 70% ↓ in AFB_2_, 100% ↓ in AFG_1_, 74% ↓ in AFB_2_	NI	Hojnik et al., 2019
AFB_1_	NI	*A. niger*	DB (Frequency: 45–250 Hz; voltage: 60–160 kV; distance: 14 mm; time: 30 sec; gas: O_2_ and N_2_; direct exposure)	95.33% Φ efficiency of AFB_1_, AFB_1_ toxicity ↓ by changes in –OCH_3_ group, double bond formation in cyclopentanone and disintegration in furan ring	C_16_H_13_O_7_ C_17_H_15_O_7_ C_15_H_13_O_7_ C_17_H_11_O_6_ C_17_H_11_O_7_ C_14_H_11_O_6_ C_16_H_11_O_6_	Zhao et al., 2023
AFB_1_	4 μL/g (Solution 50 μg/mL)	Corn (Powdered)	HVCAP [Frequency: 50 Hz; voltage: 90 kV; power: 200 W; time: 1, 2, 5, 10, 20, 30 min; gas: O_2_ (22%), N_2_ (78%); direct and indirect exposure]	62% and 82% ↓ in AFB_1_ at 1-min and 10-min	NI	Shi et al., 2017a
AFB_1_	50 μg/mL	*In vitro* (Standard AFB_1_ solution in chloroform)	HVCAP [Frequency: 50 Hz; voltage: 90 kV; power: 200 W; distance: 4.44 cm; time: 1, 2 or 5 min; gas: O_2_ (22%), N_2_ (78%); direct exposure]	76% ↓ in AFB_1_ on 5-min treatment, AFB_1_ ↓ bioactivity was to disappearance of furofuran ring’s C8-C9 double bond, Observed pathways of degradation (1) ozonolysis, (2) epoxidation, Six Φ products of AFB_1_	C_16_H_16_O_6_ C_17_H_14_O_7_ C_14_H_12_O_5_ C_14_H_10_O_6_ C_17_H_12_O_7_ C_19_H_18_O_8_	Shi et al., 2017b
AFB_1_ TAFs	10 mg/mL	*In vitro* Hazelnut (standard solutions)	DBD [Frequency: 100–150 kHz; power: 0.4–2 kW; distance: 50 mm; time: 1, 2, 4, 12 min; gas: O_2_ (0.1, 1, or 21%), N_2_]	70% ↓ of AFB_1_ and TAFs on 12 min treatment at 1 kW; 75–100% ↓ in AFB_1_; 43–100% ↓ in TAF; 0–70.9% ↓ in AFB_1_; 2.3–69.4% ↓ in TAFs	NI	Siciliano et al., 2016

*20 μL of the solution was added to each sample; AFs, aflatoxins (AFB_1_ + AFB_2_ + AFG_1_ + AFG_2_); AFB_1_, aflatoxin B_1_; AFB_2_, aflatoxin B_2_; AFG_1_, aflatoxin G_1_; AFG_2_, aflatoxin G_2_; AFM_1_, aflatoxin M_1_; TAF, total aflatoxins. CDPJ: corona discharge plasma jet; cm, centimeters; DBD, dielectric barrier discharge; I: results obtained after 1 and 5 min as well as on direct exposure; II: results obtained after 1 and 5 min as well as on indirect exposure; III: results obtained after 60 min time of exposure; IV: results obtained with 1 min time of plasma exposure; V: results obtained on 3 min time of plasma exposure; VI: results obtained after 3 and 5 min time of exposure; NOx, nitrogen oxide; O_3_, ozone; SDBD, surface dielectric barrier discharge; SDM, dielectric barrier surface micro-discharge; ↓ reduction or decrease; Φ degradation; NI, not informed.

**TABLE 3:** Summary of main outcomes from studies using cold atmospheric pressure plasma (CAP) in decontaminating other mycotoxins.

Type of mycotoxin	Initial concentration of mycotoxin	Matrix type	CAP source (process parameters)	Main outcomes	Degradation products	References
OTA and DON	OTA: 50 ng/mL DON: 10 μg/mL	Rice grain	DBD (Voltage: 25 kV; distance: 20 mm; time: 2, 4, 6, 8 min)	↓ in OTA (55%) and DON (61%), ↑ in rice protein and in prolamin contents	NI	Guo et al., 2023
DON	NI	*Fusarium*	SMD (Frequency: 7 kHz; power: 5 ± 0.15 W; distance: 3 mm; gas: O_2_, N_2_; indirect exposure)	↓ of DON in flour: 6.4% (3 min), 52.9% (6 min), 54.7% (9 min) ↓ of DON in fungal mycelia: 30.0% (3 min), 48.4% (6 min), 48.5% (9 min)	NI	Wang Y. et al., 2022
DON	2,000–2,500 μg/kg	Wheat (standard solution)	DBD (Voltage: 50 kV; time: 8 min; CO_2_, N_2_, O_2_ and atmospheric air)	83.99% DON Φ on 8 min, ↑ in wheat quality and slight ↓ whiteness in wheat powder	C_15_H_22_O_7_ C_15_H_20_O_5_ C_14_H_16_O_4_ C_15_H_21_NO_9_	Chen et al., 2022
OTA	Fungal contamination	Barley (*A. niger* and *P. verrucosum*)	DCSBD 400 [Power: 350 W; gas flow rate: 10 sL/min; gases: CO_2_ (80%) + O_2_ (20%); time: 1 or 3 min]	OTA on inoculated barley ↓ from 38.9 to 17.5 and 17 ng/g on 1 min (55% ↓) and 3 min (56.2% ↓).	NI	Durek et al., 2018
DON	200 μL/495–505 g (Solutin 50 μg/mL)	Barley grain (standard solution)	DBD (Frequency: 3,500 Hz; voltage: 0–34 kV; power: 300 W; current: 1 A; distance: 2 mm; time: 0, 2, 4, 6, 8, 10 min; gas: O_2_, N_2_)	48.9% DON ↓ (6-min), 54.4% DON ↓ (10 min)	NI	Feizollahi et al., 2020
FB_1_ ENB OTA ZEN DON	FB_1_: 2 μg/mL ENB: 0.5 μg/mL OTA: 0.5 μg/mL ZEN: 2 μg/mL DON: 10 μg/mL	*In vitro* Corn (standard solution)	DBD (Frequency: 20 kHz; voltage: 6 kV; distance: 12 mm; time: 10 min; gas: He and O_2_)	66% ↓ in FB_1_ and ENB ↓ (1.1 min half-life), OTA and ZEN ↓ (2.6 min half-life), slow ↓ in DON (74 min half-life)	AFB_1_: C_17_H_12_O_6_ C_17_H_14_O_8_ C_16_H_12_O_7_ C_15_H_12_O_7_ C_15_H_10_O_5_ C_15_H_10_O_7_ C_14_H_12_O_5_ ZEN: C_18_H_22_O_5_ C_18_H_22_O_6_ C_21_H_24_O_8_	Wielogorska et al., 2019
ZEN	l5, 10, 15 and 20 μg/mL	*In vitro* Corn kernels and flour (standard solution) Wheat kernels and flour (standard solution)	SDM (Frequency: 7 kHz; power: 10–30 W; distance: 1, 2, 3, 5 mm; indirect exposure)	96.18% ↓ in ZEN (3-min at 30 W), ↑ in wheat and corn flour glutens, 50.55% Φ of ZEN in wheat (20 min), 58.07% Φ of ZEN in corn (20 min)	C_18_H_22_O_7_ C_18_H_22_O_8_ C_18_H_22_O_6_ C_17_H_20_O_6_	Liu et al., 2024
OTA	20, 30 and 50 μg/mL (solution 100 μg/mL)	Raisin (standard solution)	GPSDP (Voltage: 0–30 kV; time: 4, 10 min; gas: Atmospheric air; indirect exposure)	62% Φ in OTA (4 min), 100% Φ of OTA (10 min), OTA Φ in non-toxic PheA, no ↑ in raisin quality attributes	C_19_H_17_ClNO_4_ C_11_H_9_ClNO_5_ C_11_H_9_NO_4_	Wang et al., 2024
OTA	100 μg/kg	Pistachio kernels (standard solution)	SDBD (Frequency: 23 kHz; voltage: 6 kV; power: 42–425 W; time: 4, 60 min; gas: O_3_ and NO_x;_ indirect exposure)	37% ↓ in OTA	NI	Laika et al., 2024
DON	100 μg/mL	DON in aqueous suspension and in powdered form	HVCAP (Frequency: 50 Hz; voltage: 70, 80, 85 kV; time: 0, 5, 10, 20 min; gas: O_2_ and N_2_; direct exposure)	99% ↓ in DON in aqueous suspension (20 min), 33% ↓ in DON in powdered state (20 min)	NI	Ott et al., 2021
OTA FB_2_	NI	*A. niger*	Plasma jet (Frequency: 25 kHz; voltage: 25 kV; distance: 12 mm; time: 6, 7.5 min; gas: Ar; indirect exposure)	No FB_2_ and OTA were detected	NI	Ouf et al., 2014
DON Trichothecenes FBs ZEN	DON and Trichothecenes: 27 μg/mL, FBs: 25 μg/mL, ZEN: 27 μg/mL	*In vitro*	SDBD (Frequency: 40 kHz; voltage: 7–10 kV; distance: 5 mm; time: 8 min; gas: O_2_, N_2_; indirect exposure)	2.7 to 0.11 mg/kg ↓ in DON; 90% ↓ in Trichothecenes, 93% ↓ in FBs, 97% ↓ in DAS, 100% ↓ of ZEN	NI	Hojnik et al., 2019
T-2 HT-2	T-2: ∼85 μg/kg HT-2: ∼87 μg/kg	Oat flour	DBD (Frequency: 25 kHz–2,5 kV; power: 6 W; time: 10, 20 and 30 min; gas: O_2_, N_2_, Ar and Air)	43% Φ of T-2 (30 min), 38% Φ of HT-2 (30 min)	NI	Kiš et al., 2020
FB_1_ DON ZEN T2	1.5 μL (solution 100 μg/mL)	*In vitro* Pure standards of FB_1_, DON, ZEN, T2	DBD (Frequency: 17 kHz; voltage: 38 kV; distance: 2 mm; power density: 4 W/cm^2^; waveform: pulsed sine; time: 5 sec, 10 sec, 20 sec, 30 sec, 1 min; gas: O_2_, N_2_)	99% Φ of FB_1_, DON, T2, ZEN (1 min)	NI	Ten-Bosch et al., 2017
PAT	100 μg/kg	Apple	DBD (Frequency: 10–50 kHz; voltage: 17–23 kV; time: 10 min; gas: O_2_, N_2_)	55% ↓ in PAT (2.5 and 8.5 min); 99% ↓ in PAT (10 min)	NI	Shirazi et al., 2025

ENB, enniatin B; DAS, diacetoxyscirpenol; DON, deoxynivalenol; FBs, fumonisins; FB_1_, fumonisin B_1_; FB_2_, fumonisin B_2_; PTA, patulin; TEN, tentoxin; OTA, ochratoxin A; T2, T2 toxin; ZEN, zearalenone; CO_2_, carbon dioxide; DBD, dielectric barrier discharge; DCSBD, diffuse coplanar surface barrier discharge; GPSDP, gas-phase surface discharge plasma; O_2_, oxygen molecule; PheA, phenylalanine; SDBD, surface dielectric barrier discharge; SMD, dielectric barrier surface micro-discharge; ↑ represents small change or affects; ↓ reduction or decrease; Φ degradation; NI: not informed.

The toxicity of AFB_1_ is attributed to the double bond between carbons 8 and 9 (C8 = C9) in furan ring, and disrupting this bond produces less toxic degraded products (Hojnik et al., 2021), depicted in Figure 4. In a study by Nguyen et al. (2022), CAP’s effect on aflatoxin M_1_ (AFM_1_) was investigated, reporting reductions across all treatments, with the most significant decrease of 60% occurring after 20 min of exposure to a high voltage generated CAP (Table 2). Similarly, Nikmaram and Keener (2022) achieved an even higher AFM_1_ degradation rate of 87% with just 4 min of CAP exposure (30–130 kV, 60 Hz) in milk. The analysis in this study indicated three TPs resulted from AFM_1_ degradation (i.e., C_15_H_11_O_7_, C_17_H_15_O_9_, and C_15_H_13_O_7_). Each TP was formed as a result from CAP induced chemical modifications in furofuran ring, particularly by the disruption of the double bond between C8 and C9, leading to a reduction in toxicity of the molecule (Nikmaram et al., 2023).

In pistachio kernels treated with ozone-based CAP, AFB_1_ and AFG_1_ were reduced by 81 and 82% within 15 min, respectively, reaching 99% after 60 min, while CAP with NOx reduced AFB_1_ and AFG_1_ by 64 and 63%, respectively (Laika et al., 2024). In wheat, CAP treatment led to 61% reduction in AFs, with specific decreases of 64, 41, 59 and 40% for AFB_1_, AFB_2_, AFG_1_ and AFG_2_, respectively (Rahnavard et al., 2024). The reduction rate of AFB_1_ increased from 62 to 82% in corn powder treated by a high voltage CAP for one to 10 min (Shi et al., 2017a). A 5-min treatment of high voltage CAP, revealed 76% degradation in AFB_1_ in standard solutions, suggesting ozonolysis and epoxidation that led to the breakdown of C8 = C9 in furofuran, which resulted in six TPs–e.g., C_16_H_16_O_6_, C_17_H_14_O_7_, C_14_H_12_O_5_, C_14_H_10_O_6_, C_17_H_12_O_7_, and C_19_H_18_O_8_ (Shi et al., 2017b; Table 2).

Hojnik et al. (2020) achieved complete AFB_1_ degradation in corn grains after 5–10 min treatment of CAP generated with SBD. Except for a negligible alteration in sample morphology, CAP application led to AFB_1_ degradation based on its four TPs such as C_17_H_15_O_7_, C_16_H_11_O_6_, C_15_H_13_O_7_, and C_14_H_10_O_6_ (Hojnik et al., 2020). In another study, a 30-s treatment of CAP with SDBD demonstrated 99% reduction in AFB_1_ and 70% in AFB_2_, while 100 and 74% reductions were observed for AFG_1_ and AFG_2_, respectively (Hojnik et al., 2019). In rice inoculated with *A. parasiticus*, CAP reduced AFs by 56.4% after 60 min with a DBD system using a mixture of O_2_, N_2_, and CO_2_. However, minor fat oxidation was noted probably because of insource generated reactive species (Zhi et al., 2023). In another study, corn inoculated with *A. flavus* and treated with CAP at intervals of 2, 4, 8, and 12 min showed progressive AFB_1_ reductions of 23.4, 41.8, 64.4, and 80.0% (Zhao L. et al., 2024). Additionally, AFB_1_ degradation by CAP in an *A. niger* cell suspension reached 95.3% within 30 s. The data indicated a disintegrated chemical structure of AFB_1_, resulting in seven TPs designated as products (Ps) in Figure 4, e.g., C_16_H_13_O_7_ (P1), C_17_H_15_O_7_ (P2), C_15_H_13_O_7_ (P3), C_17_H_11_O_6_ (P4), C_17_H_11_O_7_ (P5), C_14_H_11_O_6_ (P6) and C_16_H_11_O_6_ (P7) (Zhao et al., 2023). Previously, Wielogorska et al. (2019) obtained a 66% reduction of AFB_1_ after treatment by CAP generated with DBD demonstrated, mainly due to a high voltage in oxygen-rich environment that boosted oxygen radical and ozone formation, thereby enhancing peroxidation. This reaction primarily modified the terminal furans, with lactone and methoxy groups unchanged, indicating selective site reactivity with a net result of seven TPs such as C_17_H_12_O_6_, C_17_H_14_O_8_, C_16_H_12_O_7_, C_15_H_12_O_7_, C_15_H_10_O_5_, C_15_H_10_O_7_, and C_14_H_12_O_5_, respectively (Wielogorska et al., 2019). Similarly, wheat and rice inoculated with *A. flavus*, treated with CAP generated by CDPJ using variable frequency, current and electrodes distance, revealed remarkably a reduction of 95% of AFB_1_ of which 45 to 56% were assigned to wheat and rice samples (Puligundla et al., 2020). Moreover, DBD-based CAP for a 12 min treatment of hazelnuts showed the reduction rates between 43 and 100% for AFs, and 75 to 100% for AFB_1_ (Siciliano et al., 2016). Sakudo and Yagyu (2024) reported time-dependent reductions in AFB_1_, achieving 75.8% decreased within 1 min and up to 82.5% over 30 min of CAP exposure.

Studies have demonstrated the ability of CAP to decontaminate other types of mycotoxins (Table 3). Wang Y. et al. (2022) investigated the impact of CAP generated with SMD on *Fusarium graminearum* and observed a significant reduction in DON production across all samples evaluated, with results varying according to exposure duration. Notably, a 6-min CAP treatment resulted in over 50% reduction in DON levels. Similarly, Chen et al. (2022) reported that applying CAP generated with DBD at 50 kV to wheat for 8 min led to a DON degradation of over 62–84%. The authors mentioned that seven TPs of DON (Figure 5) were confirmed according to four molecular formulas, e.g., C_15_H_22_O_7_ (P1/1–3), C_15_H_20_O_5_ (P2), C_14_H_16_O_4_ (P3), and C_15_H_21_NO_9_ (P4/1–2) (Chen et al., 2022). Among these, C_15_H_20_O_5_ (P2), known as de-epoxy deoxynivalenol (DOM-1), was noted for its reduced toxicity compared to its parent DON molecule (Figure 5). DON’s toxicity is primarily associated with the hydroxyl (-OH) group at C3, the double bond between C9 and C10, and the sigma bond between C12 and C13 in epoxy group. Since the degradation products lack these or some of these chemical features, are considered as less harmful (Chen et al., 2022). Notably, the breakdown of the C12-C13 epoxy ring may be more prevalent in acidic environments, suggesting that food matrices with lower pH may facilitate this process (Guo et al., 2023). In the same study, it was also demonstrated that CAP generated with DBD effectively reduces the levels of both DON and OTA. The highest reduction was obtained after 8 min of treatment, reaching 61 and 56% reduction rates for DON and OTA, respectively (Guo et al., 2023). Regarding OTA, CAP treatment promotes the hydrolysis of the amide bond, thus resulting in the formation of less toxic compounds like phenylalanine (C_9_H_9_NO_2_) and ochratoxin-α (OT-α: P2) (Figure 6). It is also notable that ozone generated by CAP can oxidize the chlorinated ring in the OTA moiety, which leads to its breakdown into amino acids or free chlorine, further contributing to detoxification (Guo et al., 2023; Wang L. et al., 2022). Wang et al. (2024) demonstrated the effectiveness of CAP generated with GPSDP in degrading OTA in raisin, achieving 62% degradation at a four-min treatment and reaching 100% with a 10-min treatment (Table 3), without affecting matrix quality. Three OTA degradation products such as C_19_H_17_ClNO_4_ (P1), C_11_H_9_ClNO_5_ (P2) and C_11_H_9_ClO_4_ (P3) were identified (Figure 6), likely resulting from ozone, peroxides, and free radicals, as detailed elsewhere (Guo et al., 2023; Wang L. et al., 2022; Wang et al., 2024).

In corn containing six mycotoxins and treated with CAP using a DBD system, Wielogorska et al. (2019) observed 60–66% reductions of AFB_1_ and FB_1_ within 10 min. The study led to the identification of TPs only for AFB_1_ (e.g., C_17_H_12_O_6_, C_17_H_14_O_8_, C_16_H_12_O_7_, C_15_H_12_O_7_, C_15_H_10_O_5_, C_15_H_10_O_7_ and C_14_H_12_O_5_) and ZEN (e.g., C_18_H_22_O_5_, C_18_H_22_O_6_ and C_21_H_24_O_8_). Helium plasma enhanced the degradation efficiency when compared with mixed nitrogen-oxygen, primarily due to increased oxygen radical formation and a preference for peroxidation reactions (Wielogorska et al., 2019). A three-min CAP treatment using SDM at 30 watts achieved 96.2% degradation of ZEN in corn and wheat kernels, as well as their flour. However, individual treatments of wheat and corn alone over 20 min showed ZEN reductions of 50.5–58.1%, with no significant changes to gluten in either matrix. Four ZEN degradation products, presented as C_18_H_22_O_7_ (P1), C_18_H_22_O_8_ (P2), C_18_H_22_O_6_ (P3/1–2) and C_17_H_20_O_6_ (P4) in Figure 7, were identified, primarily from oxidative cleavage of the olefinic C11 = C12 bond, following well-explored pathways (Liu et al., 2024). Key factors in ZEN degradation included irradiation distance, treatment time, RONS, and free radical oxidation.

The use of CAP for mycotoxin decontamination has primarily focused on the widely consumed cereals, grains, and their derivatives, with limited attention given to perishable products like fruits and vegetables. Research on perishable matrices has mainly addressed microbial control and shelf-life extension, leaving the potential for mycotoxin mitigation largely unexplored (Yinxin et al., 2022; Pinela and Ferreira, 2017; Yarabbi et al., 2023). A recent study highlighted the effectiveness of CAP generated with DBD in degrading PAT in fresh-cut apple slices while simultaneously enhancing their quality attributes (Shirazi et al., 2025). Treatment with 23 kV for 2.5 and 8.5 min gave 55 to 99% reduction of PAT to the undetectable levels, demonstrating the importance of high voltage and optimized exposure time. The degradation was attributed to ROS produced during plasma discharge, which facilitated oxidative breakdown into seven TPs (Figure 8) including C_10_H_23_O_4_ (P1), C_8_H_16_O_3_ (P2), C_5_H_10_O_2_ (P3), C_4_H_8_O_2_ (P4), C_3_H_6_O_2_ (P5), C_3_H_8_O_3_ (P6), and C_4_H_8_O_3_ (P7) (Shirazi et al., 2025; Xue et al., 2021). These findings highlight the dual benefits of CAP in mitigating mycotoxins and enhancing food quality. The demonstrated efficacy of CAP for perishable products like fresh-cut fruits provides a foundation for broader applications and indicates the potential for further research and development in food safety and preservation strategies.

The efficiency of CAP in degrading different mycotoxins and reducing their toxicity depends on its ability to induce structural modifications in the target mycotoxin molecules, particularly at the molecular sites responsible for their toxicity. Degradation is confirmed only when less toxic molecules are generated. However, despite the significance of understanding the toxicity of degradation products, only 7 out of the 30 studies reviewed provided data on the by-products of the given toxins degraded by the CAP technology. In conformity with these results, the TPs mentioned and those newly emerging should be comprehensively studied for their potential toxicities and risks. While challenging, accurately measuring the maximum number of detectable TPs requires highly sensitive methods, likely the use of both high-resolution mass spectrometry (HRMS) and low-resolution MS, for inclusive proof of identities, validation, and quantification of all novel TPs. To ensure compliance with the guidelines set by the World Health Organization (WHO) and the Food and Agriculture Organization (FAO), both *in vitro* and *in vivo* studies are essential to assess the overall toxicological profiles of the TPs generated by CAP treatments.

## 6 Effects of cold atmospheric plasma on the food matrix

Assessing the impact of CAP on food products is crucial, particularly regarding their physicochemical, sensory, and nutritional properties. Research indicates that CAP treatment does not significantly alter the nutritional composition of food matrices. Studies on raw materials like rice and wheat, as well as processed products like wheat flour, show that starch content remains largely unaffected by CAP exposure (Guo et al., 2023; Chen et al., 2022; Zhi et al., 2023; Zhao L. et al., 2024).

Regarding the sensory attributes, specifically color, CAP treatment of rice revealed no significant changes in its characteristic appearance (Zhi et al., 2023). In the case of milk, however, Nikmaram and Keener (2022) observed a non-perceptible color change in skim milk, while a slight but perceptible yellowing occurred in whole milk. This yellowing effect is likely due to the oxidation of fats and proteins induced by CAP which is a source of highly reactive species (Ali et al., 2023b; Segat et al., 2015).

The limited impact of CAP on the food matrices may also be attributed to its inability to penetrate deeply into the product. For example, in rice, the presence of husks reduces CAP’s effectiveness (Guo et al., 2023). Similar results were observed in corn kernels, where analytical methods such as X-ray photoelectron spectroscopy (XPS) and secondary ion mass spectrometry (SIMS) demonstrated that 8 min treatment with air plasma generated with SBD led to the slight oxidation of corn kernel surface, while scanning electron microscopy (SEM) showed no significant differences between CAP-treated and control samples (Hojnik et al., 2020).

CAP treatment can have varying effects on the food protein composition. While there were significant differences in specific proteins, such as prolamin and globulin, between the CAP-treated and control samples, the overall protein content remained unaffected. These findings are consistent with those of Zhi et al. (2023), where even prolonged exposure to CAP (60 min) did not result in significant changes. Regarding fatty acid (FA) content, CAP treatment was observed to increase the number of FA types over time, generating 3 to 5 new FAs. However, the primary FA components in wheat showed no significant difference compared to the control, suggesting that CAP does not alter the total FA content (Guo et al., 2023). In contrast, Zhi et al. (2023) reported a significant upsurge of 275.3% in free FA content in rice, indicating that CAP can indeed influence such composition. These findings collectively indicate that the application of CAP using different system parameters and exposure times had none or negligible qualitative effects on food matrices, as described for various items including dairy products (milk), and cereal grains (e.g., rice, wheat, corn).

## 7 Challenges of cold atmospheric plasma application in the food industry

CAP is an emerging technology in food mycotoxin decontamination due to its high efficiency, environmentally friendly nature, and dual functionality in degrading toxins and inhibiting the growth of toxigenic microorganisms (Marshall et al., 2020; Alizadeh et al., 2021; Ouf et al., 2014; Wang et al., 2020). Despite these advantages, several challenges obstruct its industrial application. Scalability remains a major challenge, while the DBD model, as the easiest of all possible options and most used in research, presents significant obstacles for industrial implementation (Neuenfeldt et al., 2023; Guo et al., 2023). The lack of standardized range for key parameters such as discharge frequency, voltage, and power, also complicate its optimization for large-scale use (Neuenfeldt et al., 2023). Future advancements should prioritize the development of modular, scalable CAP systems and the establishment of standardized operational parameters to broaden CAP’s applicability while achieving consistent and reproducible outcomes. The cost of CAP equipment, energy demands, and limited penetration of reactive species in complex food matrices remains another barrier to be addressed (Chakka et al., 2021). Designing cost-effective, energy-efficient CAP devices integrated with mechanisms like agitation systems or rotating conveyor belts could enhance reactive species distribution, improve decontamination efficiency, and facilitate industrial applications.

The literature highlights a well-understood degradation pathway for mycotoxins like AFB_1_ and DON. However, there is limited knowledge regarding the degradation products and cytotoxicity of other important mycotoxins, such as AFM_1_ and OTA (Hojnik et al., 2020; Deng et al., 2020). Additionally, research on the effects of CAP on the physicochemical, nutritional, and sensory properties of food matrices is scarce, underscoring the need for further investigation in this area while maximizing CAP efficiency (Guo et al., 2023). Taken together, overcoming these obstacles will need interdisciplinary collaboration across engineering, toxicology, and food science. Such efforts will be pivotal in advancing CAP as a sustainable, scalable, and reliable technology in the food sector.

## 8 Concluding remarks and future perspectives

This systematic review confirms the effectiveness of CAP in degrading mycotoxins such as AFB_1_ and DON. The extensive knowledge of the degradation compounds and their reduced toxicity underscores CAP’s potential in food safety applications. However, the review highlights a significant gap in research regarding the cytotoxicity of these degradation by-products. Future studies should focus on the cytotoxicological assessment of by-products from the breakdown of a broader range of mycotoxins, including AFs, FBs, OTs, ZEN, DON, T-2, HT-2 and PAT. CAP also shows promise in preventing mycotoxin contamination by inhibiting the growth of toxigenic fungi. The technology effectively targets fungal spores and damages the DNA responsible for mycotoxin biosynthesis, offering a dual approach to both degrading existing mycotoxins and preventing their formation. Despite its potential, the industrial application of CAP faces several challenges, particularly regarding the practical viability of large-scale implementation. Key obstacles include the need for more robust equipment design and the optimization of operational parameters, as there is currently no consensus on the best practices for these variables. The literature suggests that CAP has a minimal impact on the nutritional and sensory qualities of food, making it an attractive option for the food industry. However, research in this area remains limited, highlighting the need for more studies to fully understand the CAP efficacy on different food matrices. As a cutting-edge method for mycotoxin decontamination, addressing these research gaps is crucial for advancing the development and industrial applicability of CAP. Further exploration will enhance the efficiency of decontamination processes, ultimately contributing to the production of safer food products on a larger scale.

## Data Availability

The original contributions presented in this study are included in this article/supplementary material, further inquiries can be directed to the corresponding author.
